# Place of death associated with types of long-term care services near the end-of-life for home-dwelling older people in Japan: a pooled cross-sectional study

**DOI:** 10.1186/s12904-020-00622-0

**Published:** 2020-08-09

**Authors:** Kazuhiro Abe, Atsushi Miyawaki, Yasuki Kobayashi, Taeko Watanabe, Nanako Tamiya

**Affiliations:** 1 Department of Public Health, Graduate School of Medicine, The University of Tokyo, 7-3-1 Hongo, Bunkyo-ku, Tokyo, 113-0033 Japan; 2 Health Services Research and Development Center, University of Tsukuba, 1-1-1 Tenno-dai, Tsukuba, Ibaraki, 305-8575 Japan; 3 Department of Health Services Research, Faculty of Medicine, University of Tsukuba, 1-1-1 Tenno-dai, Tsukuba, Ibaraki, 305-8575 Japan

**Keywords:** Long-term care insurance, Place of death, In-home services, Day services, Short-stay services

## Abstract

**Background:**

Many older people wish to die at home. However, there is still a huge gap between the place where older adults wish to die and the place where they, in fact, do die. We aimed to assess the association between each type of long-term care (LTC) services that home-dwelling older individuals utilized at their end of life and place of death.

**Methods:**

A pooled cross-sectional study at the point of death was used for the analysis. Participants included beneficiaries of long-term care insurance in Japan, aged 65 years and above, who passed away between January 2008 and December 2013, excluding those who died due to external factors and those who were using residential services at their time of death. We conducted a multivariate Poisson regression analysis with robust standard errors adjusting for potential confounders and examined the association between the use of each type of LTC service for home-dwelling recipients, including in-home services, day services, and short-stay services, with the interaction terms being time of death (exposure) and home death (outcome). We calculated the adjusted probability of home deaths for each combination pattern of LTC services for home-dwelling recipients using standard marginalization.

**Results:**

We analyzed 2,035,657 beneficiaries. The use of in-home services, day services, and short-stay services were associated with an increased probability of home deaths; the incident rate ratio (IRR) was 13.40 (with a 95% confidence interval (CI): 13.23–13.57) for in-home services, the IRR was 6.32 (6.19–6.45) for day services, and the IRR was 1.25 (1.16–1.34) for short-stay services. Those who used day or short-stay services with in-home services exhibited a higher probability of home deaths than those who used only day or short-stay services.

**Conclusions:**

We demonstrated that home-dwelling older persons who used LTC services near end-of-life had a higher probability of home deaths as compared to those who did not. Our findings can clarify the importance of providing and integrating such services to support care recipients who wish to die at home as well as for the benefit of their informal caregivers.

## Background

Many older people wish to die in the comfort of their homes [1, 2]. Dying individuals prefer to spend their last days in a familiar environment, wherein they are surrounded by loved ones [2]. Moreover, the satisfaction of family members who provide care to an older person would be improved if the environment enabled older people to take care of themselves toward the end of their lives in their preferred place [3]. However, there is still a huge gap between the place where older adults wish to die and the place where they, in fact, do die. For example, in Japan, a nationwide survey conducted in 2012 revealed that older people over the age of 55 were more likely to expect to spend their life’s last moments at home instead of hospitals and facilities; 54.6% wanted to die at home [4]. However, the proportion of home deaths had reduced to 13.2% of all deaths in 2017, and the proportion of hospital deaths increased to 73.0% in the same year [5]. Given these gaps between the ideal and reality regarding place of death, it is essential to investigate the factors promoting the ability to experience a home death.

There are a few reasons why dying older people could not stay at home until their deaths. First, without social or care support, dying older individuals cannot maintain their dignity and an independent everyday life routine according to their level of ability [6]. At the end of life, in particular, the burden of care is among one of the most serious issues faced by informal caregivers, which sometimes causes care recipients to sacrifice staying at home [7]. Formal long-term care (LTC) services, defined by the World Health Organization as “The activities undertaken by others to ensure that people with or at risk of a significant ongoing loss of intrinsic capacity can maintain a level of functional ability consistent with their basic rights, fundamental freedoms and human dignity,” are considered to support the daily lives of care recipients and to relieve caregivers’ burden [8, 9]. Therefore, the appropriate use of LTC services for home-dwelling older people may help a dying older person and his/her caregivers continue home care until the individual’s death.

According to previous studies, home deaths of patients have been positively associated with the use of in-home services or multidisciplinary home palliative care provided by medical professionals such as physicians and nurses [10–13]. Most reviewed studies reported a higher proportion of home deaths for older cancer patients who used in-home services as compared with those who did not [10–12]. Furthermore, a 2013 Cochrane review reported the effect of multidisciplinary home palliative care provided by physicians, nurses, and medical social workers on home deaths, demonstrating that such intervention increases the probability of home deaths [13]. However, although these studies addressed the in-home services provided by *medical* professionals, the association of in-home, day, and short-stay services provided by *non-medical* professionals—such as care workers—with the care recipients’ place of death remains virtually unknown. Most recently, a study demonstrated a positive association between the in-home service provided by care workers and home deaths; however, it did not focus on LTC services other than in-home services for care [14]. Furthermore, some ecological studies have reported that good accessibility to LTC services for home-dwelling recipients, including in-home, day, and short-stay, of the living municipality was associated with a higher proportion of home deaths [15–17]. However, at the individual level, the association of these LTC services for home-dwelling recipients with home deaths still remains unclear.

In this context, the present study aims to assess the association between the use of LTC services for home-dwelling recipients and recipients’ place of death at the end of life, using nationwide LTC insurance claims data in Japan.

### Overview of medical and LTC services for terminally ill older people in Japan

The Japanese government introduced the Long-Term Care Insurance (LTCI) in 2000 in the form of a social insurance system with compulsory participation. LTCI provides LTC services through in-kind benefits to those aged 40 years and above with intractable disease and those aged 65 years and above when they are eligible for receiving care. The copayment for using LTC services is between 10 and 30% of expenditures, and the proportion of copayment is decided by the care recipients’ income [18]. The upper limit of recipients’ budget is set according to the seven stages of degree of care needed. It includes two stages that require support for the instrumental activities of daily living (IADL) (support levels 1 and 2) and five that require LTC for activities of daily living (ADL) (care levels 1–5) (a higher level indicates a higher need for care) [19]. When those who wish to receive LTCI apply to their municipal government, a qualified surveyor conducts an on-site survey consisting of 73 survey items to analyze the physical and cognitive functioning of the applicants. By referring to the results of an objective, computer-assisted evaluation from the surveyor’s findings and the opinion of a physician in charge, The Needs Assessment Review Committee, which consists of approximately five health and welfare professionals, decides the degree of care needed for each applicant [20].

Japan’s LTC services are roughly divided into two categories according to the place where service-recipients live. One category is services for home-dwelling recipients, which include (i) in-home services for care, nursing, medical support, bathing, rehabilitation, and renting of welfare equipment such as wheelchairs and care beds; (ii) day services for care and rehabilitation; and (iii) short-stay services for care and care with medical services. The other category covers services for residents at LTC facilities and qualified nursing homes. A trained and qualified care manager in charge coordinates what kind of LTC services should be used and are applicable for each beneficiary in the upper budget of each stage, in consultation with care recipients and their families. Table 1 shows the primary service providers, places, and types of services [21–28]. In Japan, the qualifications of physicians, nurses, pharmacists, therapists, dieticians, dentists, and dental hygienists among the service providers in Table 1 are regulated by healthcare laws. By contrast, care workers and specialized consultees for renting welfare equipment, who also played a major role in the provision of LTC services, are subject to LTC-related laws. Care workers differ from medical professionals, such as nurses, in that they are not necessarily required to obtain any special qualifications, although they are encouraged to take initial training.

**Table 1:** Primary providers, places, and services in each type of LTC^a^ services covered by Japan’s LTCI^b^

Type of LTC	Provider	Place	Service
1. Services for home-dwelling recipients			
(i) In-home services			
for care	Care workers	Recipients’ homes	Care workers regularly assist in caring for recipients with essential daily routine activities.
for nursing	Nurses or therapists	Recipients’ homes	Nurses or therapists regularly provide nursing or rehabilitation to care recipients.
for medical support	Physicians, nurses, pharmacists, dieticians, dentists, or dental hygienists	Recipients’ homes	Medical professionals provide information on the recipients’ medical condition and give medical advice to care workers, care recipients, or informal caregivers with referral forms or by parol.
for bathing	Nurses and care workers	Recipients’ homes	Nurses and care workers regularly assist with bathing to care recipients.
for rehabilitation	Therapists	Recipients’ homes	Therapists regularly offer rehabilitation to care recipients.
for renting welfare equipment	Specialized consultees	Recipients’ homes	Consultees design a plan for renting welfare equipment, including wheelchairs, care beds, devices for postural changes on the bed, arm rails, walkers, sticks, sensing devices against wandering, and transfer aids. Moreover, they even lend and maintain them.
(ii) Day services			
for care	Care workers and nurses	Day service facilities	Care recipients regularly stay at the day service facilities during the daytime to prevent the social isolation of care recipients and reduce the burden of informal caregivers.
for rehabilitation	Therapists, nurses, and care workers	LTC health facilities, hospitals, or clinics	Care recipients regularly receive rehabilitation during the daytime to improve and maintain their physical and psychological condition.
(iii) Short-stay services			
for care	Care workers	LTC welfare facilities	Care recipients stay at LTC welfare facilities for one day to one month to reduce the burden of informal caregivers and maintain the physical and psychological conditions of the recipients.
for care with medical services	Medical professions and care workers	LTC health or medical facilities, hospitals, and clinics with beds	Care recipients stay at the facilities with medical care for one day to one month to reduce the burden of informal caregivers and maintain the medical condition of recipients.
2. Services for residents at LTC facilities and nursing homes			
at LTC welfare facilities	Care workers and nurses	LTC welfare facilities	The facilities provide a place for recipients who have difficulties in living at home due to a high degree of care needed.
at LTC health facilities	Care workers, physicians, and nurses	LTC health facilities	The facilities provide a place for rehabilitation to care recipients.
at LTC medical facilities	Physicians and nurses	LTC medical facilities	The facilities offer an LTC with medical treatments to care recipients. Hospitals and clinics with beds run the facilities.
at qualified nursing homes	Care workers and nurses	Nursing homes	Qualified nursing homes, which fulfill the required government facility criteria, provide a place for recipients who have difficulty living at home.

^a^ Long-term care

^b^ Long-term care insurance

Terminally ill older people with medical needs can also use the outpatient, inpatient, and in-home medical care that is provided by hospitals and clinics, which is covered by universal social health insurance in Japan. Although when physicians order a visit based on the severity and urgency of a patient’s condition, nurses can provide in-home medical care for the short-term under health insurance, regular in-home care by nurses is preferentially covered by LTCI. In addition, although admission to palliative care beds is covered by health insurance, only 0.4% of all beds in Japan were registered as palliative care beds in 2013 [29, 30]. Therefore, older people are likely to end their lives in hospitals, but in other types of beds.

## Methods

### Participants, study design, and data

A pooled cross-sectional study around the time of the participants’ death was deployed. The participants included LTCI beneficiaries in Japan, aged 65 years and above, who had passed away between January 2008 and December 2013, excluding those whose deaths were caused by external factors and individuals who used residential and municipality-based services at their time of death. The details of these exclusion conditions are described as follows.

With the official approval to use the secondary data from the Statistics and Information Department of the Ministry of Health, Labour and Welfare (MHLW) under approval number 1130–1, we utilized the individual data from the Survey of Long-term Care Benefit Expenditures and the death records from the Vital Statistics of Japan for the years between 2006 and 2017 [31]. The Survey of Long-Term Care Benefit Expenditures records monthly panel data regarding the eligibility period, month and year of birth, gender, degree of care needs, LTC service usage, and living municipal codes of LTCI beneficiaries throughout Japan. The death records in the Vital Statistics database are built using the death certificates issued by physicians across the country, wherein the following data are recorded: age of death, gender, month and year of birth, presence of spouse at death, underlying cause of death, place of death, date of death, and living municipal code. We linked both these administrative databases by a deterministic linkage, using four items of demographic data to identify death for each LTCI beneficiary; municipality of residence, gender, month and year of birth, and date of death (as all individual data were anonymized by the MHLW, we could not identify a specific individual by a common variable). Although the date of death is not included in the survey of Long-Term Care Benefit Expenditures, the eligibility for LTCI expires exactly 1 day after the date of death on the death certificate. Thus, we matched the data indicating the date of the recipients’ death to 1 day before LTCI eligibility expiry. We first extracted 7,367,009 participants with expired LTCI eligibility and 14,701,083 who passed away in Japan from 2006 to 2017. Among them, we excluded 2.7% of participants with expired LTCI eligibility in the Survey of Long-term Care Benefit Expenditures and 0.3% of participants who passed away in the death records, as the four identification variables were overlapped owing to the lack of the patient’s exact date of birth. Then, we conducted the deterministic linkage, and 16.2% of those with expired LTCI eligibility that were unmatched were excluded from the analysis as those who moved to other municipalities. During the study period (January 2008–December 2013), the remaining 2,887,396 participants were considered to be LTCI beneficiaries aged 65 years and older who had passed away.

We excluded 84,426 recipients who died owing to external factors such as by accident or suicide (V01-Y89 on the International Classification of Diseases 10 (2003)) because the home deaths envisioned by many older people were not considered to be caused by such an underlying cause of death. In addition, we excluded 666,897 residents of residential services covered by LTCI during/in the month of death because the LTCI system mandates that only recipients with a high degree of care needed can enter LTC facilities and that most LTC services for home-dwelling recipients cannot be used while a recipient is in LTC facilities. Moreover, the residents were not analytically comparable because less than 1% of them died at home. Ninety-nine thousand and four hundred and twenty-four users of municipality-based services, which were provided by each municipality at its own discretion since 2006, were excluded because of the limited number of provider offices during the study period and the heterogeneity of the services provided between municipalities. Although no missing values could be attributed to the administrative data, there were 992 recipients with unknown codes indicating the beneficiaries’ degree of care needed and the presence of a spouse at the time of death. These recipients were also excluded from the study. Finally, we analyzed the remaining 2,035,657 participants that could use LTC services for home-dwelling recipients. We assumed that these participants needed care and died during the study period because Japan’s LTCI requires compulsory participation and older people can use LTC services after the determination of the degree of care needed whenever they require care.

### Outcome and exposures

The primary outcome was an indicator of home deaths of recipients as identified from the Vital Statistics data. Other places of death included hospitals, clinics with beds, LTC facilities, nursing homes, and others such as day services’ facilities or outdoors.

The chief exposures were indicators of whether a participant used each type of LTC services for home-dwelling recipients, namely, (i) in-home service, (ii) day service, and/or (iii) short-stay service, at least once during/in the month of death and their interaction terms. Moreover, to explore the association between the items of each type of LTC service for home-dwelling recipients with home deaths, we used each detailed LTC service explained in Table 1, including (i) in-home services for care, nursing, medical support, bathing, rehabilitation, and renting welfare equipment, (ii) day services for care and rehabilitation, and (iii) short-stay services for care and care with medical services, during/in the month of death.

### Covariates

Referring to the determinants of place of death found in previous studies, [10–12] we adjusted the following covariates: the recipients’ age at the time of death; gender; presence of spouse (presence, unmarried, bereavement, and divorce); underlying cause of death (cancer (C00-C97), cerebrovascular disease (I60-I69), cardiovascular disease (I01, I020, I05-I09, I20-I25, I27, and I30-I52), senility (R54), and pneumonia (J12-J18)); year of death; degree of care needed; and dummy variables of secondary medical areas.

The underlying cause of death rested on the International Classification of Diseases 10th Revision (2003) [32]. We used dummy variables for degree of care needed and divided them into the following three categories: a low degree, including levels 1 and 2 requiring IADL support; a moderate degree, including levels 1 and 2 requiring support for a part of the ADL, and the high degree, including levels 3 through 5 requiring support for all ADLs [14]. Secondary medical areas are defined by the Japanese government to ensure patient access to inpatient services. Most of them are formed from adjacent municipalities. We assumed that the adjustments in the dummy variables of secondary medical areas would resolve the effect of recipients’ residences, such as the difference of availabilities of hospitals, clinics, LTC facilities, or nursing homes.

### Statistical analyses and ethics

We conducted a Poisson regression analysis with robust standard errors (SE), instead of logistic regression analysis because the probability of home deaths—the outcome of this study—was predicted not to be low enough to regard the odds ratios as approximately equal to the incident rate ratios [33]. The incident rate ratio (IRR), robust SE, *p*-value, and 95% confidential interval (CI) were shown. The probabilities of home deaths with the use of each type of LTC services for home-dwelling recipients were calculated with delta-method SE using the models derived from the Poisson regression [34]. All data management and analyses were conducted using Stata 15 MP (College Station, TX; StataCorp LLC.). We considered a *p*-value < 0.05 to be statistically significant.

This research was conducted with permission from the Ethics Review Committees of the University of Tokyo (Approval No. 11070–2) and the University of Tsukuba (Approval No. 1324).

## Results

We analyzed 2,035,657 LTCI beneficiaries who passed away during the period of the study. This number represented 32.8% of all deaths of individuals aged over 65 years in Japan. The proportion of home deaths to all deaths was approximately 14% annually, and the number of older people who were ending their lives in LTC facilities and qualified nursing homes was increasing (Table 2 and Additional Table 1). In terms of the underlying cause of death in Table 2, a higher proportion of those who died at home passed away owing to cardiovascular issues and senility, while a higher proportion of those who died in hospitals and clinics had pneumonia. In addition, the number for no use of LTC services for home-dwelling recipients during/in the month of death was 1,098,458 (54.0%) of all participants. Conversely, among those who died at home, only 25,202 (8.9%) participants had no use of the services.

**Table 2:** Characteristics of LTC^a^ beneficiaries who passed away in different places in Japan

		Place of death			
			Other places		
	Total	Home	Hospitals and clinics	LTC facilities^b^ and qualified nursing homes	Others^c^
**Number of death, n (%)**	2,035,657 (100)	282,812 (100)	1,721,399 (100)	18,522 (100)	12,924 (100)
**Number of death by the year, n (%)**					
**2008**	316,830 (15.6)	45,079 (15.9)	268,426 (15.6)	1730 (9.3)	1595 (12.3)
**2009**	316,871 (15.6)	43,889 (15.5)	269,295 (15.6)	1976 (10.7)	1711 (13.2)
**2010**	334,882 (16.5)	45,777 (16.2)	284,717 (16.5)	2509 (13.5)	1879 (14.5)
**2011**	350,997 (17.2)	47,818 (16.9)	297,725 (17.3)	3190 (17.2)	2264 (17.5)
**2012**	372,326 (18.3)	52,566 (18.6)	312,764 (18.2)	4286 (23.1)	2710 (21.0)
**2013**	343,751 (16.9)	47,683 (16.9)	288,472 (16.8)	4831 (26.1)	2765 (21.4)
**Median (IQR**^**d**^**) age, years**	85 (79, 90)	86 (80, 92)	84 (79, 90)	88 (83, 93)	86 (81, 92)
**Gender, n (%)**					
**Male**	997,574 (49.0)	126,307 (44.7)	858,742 (49.9)	7268 (39.2)	5257 (40.7)
**Female**	1,038,083 (51.0)	156,505 (55.3)	862,657 (50.1)	11,254 (60.8)	7667 (59.3)
**Degree of care need, n (%)**					
**Low**	175,819 (8.6)	24,269 (8.6)	150,450 (8.7)	357 (1.9)	743 (5.7)
**Moderate**	527,942 (25.9)	69,688 (24.6)	453,521 (26.3)	2356 (12.7)	2377 (18.4)
**High**	1,331,896 (65.4)	188,855 (66.8)	1,117,428 (64.9)	15,809 (85.4)	9804 (75.9)
**Presence of a spouse, n (%)**					
**Present**	908,572 (44.6)	118,531 (41.9)	781,326 (45.4)	4916 (26.5)	3799 (29.4)
**Unmarried**	64,827 (3.2)	7838 (2.8)	55,166 (3.2)	1140 (6.2)	683 (5.3)
**Bereavement**	979,905 (48.1)	146,488 (51.8)	814,331 (47.3)	11,390 (61.5)	7696 (59.5)
**Divorce**	82,353 (4.0)	9955 (3.5)	70,576 (4.1)	1076 (5.8)	746 (5.8)
**Underlying cause of death, n (%)**					
**Cancer**	570,699 (28.0)	80,674 (28.5)	483,663 (28.1)	3154 (17.0)	3208 (24.8)
**Cardiovascular**	331,027 (16.3)	70,576 (25.0)	253,270 (14.7)	4260 (23.0)	2921 (22.6)
**Pneumonia**	264,472 (13.0)	11,526 (4.1)	250,787 (14.6)	1384 (7.5)	775 (6.0)
**Cerebrovascular**	221,564 (10.9)	27,378 (9.7)	190,681 (11.1)	2105 (11.4)	1400 (10.8)
**Senility**	96,846 (4.8)	37,952 (13.4)	54,396 (3.2)	2867 (15.5)	1631 (12.6)
**Others**	551,049 (27.1)	54,706 (19.3)	488,602 (28.4)	4752 (25.7)	2989 (23.1)
**Use of the LTC services for home-dwelling recipients**^**e**^**, n (%)**					
** No use**	1,098,458 (54.0)	25,202 (8.9)	1,071,575 (62.3)	617 (3.3)	1064 (8.2)
** In-home services**	807,785 (39.7)	240,542 (85.1)	541,951 (31.5)	14,840 (80.1)	10,452 (80.9)
**for care**	397,297 (19.5)	113,952 (40.3)	264,960 (15.4)	11,506 (62.1)	6879 (53.2)
**for nursing**	232,192 (11.4)	89,635 (31.7)	135,650 (7.9)	3739 (20.2)	3168 (24.5)
**for medical support**	170,541 (8.4)	80,216 (28.4)	78,873 (4.6)	6731 (36.3)	4721 (36.5)
**for bath**	140,820 (6.9)	69,864 (24.7)	68,364 (4.0)	1025 (5.5)	1567 (12.1)
**for rehabilitation**	21,958 (1.1)	5936 (2.1)	15,454 (0.9)	316 (1.7)	252 (1.9)
**for renting welfare equipment**	669,750 (32.9)	211,148 (74.7)	438,150 (25.5)	11,719 (63.3)	8733 (67.6)
** Day services**	385,573 (18.9)	81,188 (28.7)	294,447 (17.1)	5714 (30.8)	4224 (32.7)
**for care**	312,656 (15.4)	68,173 (24.1)	235,631 (13.7)	5079 (27.4)	3773 (29.2)
**for rehabilitation**	84,424 (4.1)	14,954 (5.3)	68,198 (4.0)	760 (4.1)	512 (4.0)
**Short-stay services**	148,701 (7.3)	30,310 (10.7)	110,636 (6.4)	5873 (31.7)	1882 (14.6)
**for care**	127,525 (6.3)	26,084 (9.2)	94,630 (5.5)	5046 (27.2)	1765 (13.7)
**for care with medical services**	23,394 (1.1)	4620 (1.6)	17,677 (1.0)	953 (5.1)	144 (1.1)
**Combined use of each type of LTC services for home-dwelling recipients**^**e**^**, n (%)**					
**In-home services and day services**	280,256 (13.8)	64,871 (22.9)	207,022 (12.0)	5052 (27.3)	3311 (25.6)
**In-home services and short-stay services**	107,377 (5.3)	27,023 (9.6)	75,939 (4.4)	3164 (17.1)	1251 (9.7)
**Day services and short-stay services**	83,338 (4.1)	19,194 (6.8)	61,680 (3.6)	1777 (9.6)	687 (5.3)
**In-home, day service, and short-stay service**	66,111 (3.2)	16,658 (5.9)	47,431 (2.8)	1471 (7.9)	551 (4.3)

^a^Long-term care

^b^LTC facilities include LTC welfare, health, and medical facilities

^c^Others indicate the place of death except for home, hospitals, clinics with beds, LTC facilities, and qualified nursing homes. (e.g., day services’ facilities or outdoors)

^d^Interquartile range

^e^LTC services for home-dwelling recipients were used during/in the month of death

In Table 3, the usage of all types of LTC services for home-dwelling recipients was associated with an increased probability of home death; the IRR for in-home services was 13.40 (with a 95% CI; 13.23–13.57); for day services was 6.32 (6.19–6.45); and that for short-stay services was 1.25 (1.16–1.34). The quadratic interaction terms were negatively associated with home death, and the cubic interaction term was positively associated with it. The results with covariates have been demonstrated in Additional Table 2.

**Table 3:** Association between home deaths and use of LTC^a^ type for home-dwelling recipients^b^ (*n* = 2,035,657)

Type of LTC^c^	Incident rate ratios	Robust SE^d^	*p*-value	95% Confidential Interval	
No use	Reference				
In-home services	13.40	0.089	< 0.001	13.23	13.57
Day services	6.32	0.066	< 0.001	6.19	6.45
Short-stay services	1.25	0.045	< 0.001	1.16	1.34
In-home and day services	0.10	0.001	< 0.001	0.10	0.11
In-home and short-stay services	0.53	0.020	< 0.001	0.49	0.57
Day and short-stay services	0.72	0.030	< 0.001	0.67	0.78
In-home, day, and short-stay services	2.14	0.091	< 0.001	1.96	2.32

^a^Long-term care

^b^Poisson regression analysis, which was conducted to examine the association between the use of LTC service type for home-dwelling recipients and home death, adjusts for recipients’ age at time of death, gender, presence of spouse, underlying cause of death, year of death, degree of need for care, and secondary medical area dummy variables. Exponentiated coefficients were shown as incident rate ratios

^c^The types of LTC services were used by recipients at least once during/in the month of death. Another service type was not used

^d^Standard errors

**Fig. 1:**
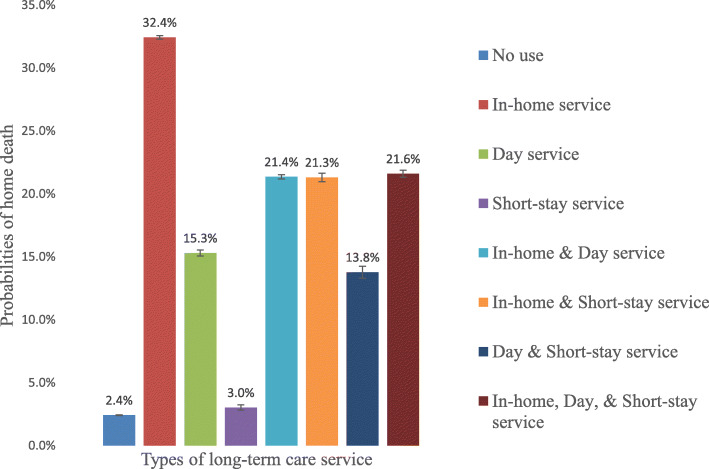
Probability of home deaths with use of each type of LTC services for home-dwelling recipients. The figure shows the adjusted probability of home death, estimated using marginal standardization (also known as predictive margins or margins of responses). Poisson regression with robust standard errors was applied, adjusting for the recipients’ age at the time of death, gender, presence of a spouse, underlying cause of death, year of death, degree of care needed, and the dummy variables of secondary medical areas. Error bars display the 95% confidence intervals. Even when we considered multiple comparisons using the Bonferroni method, the probability of home deaths for the eight categories was statistically significant for all the pairwise comparisons (28 pairs). The other service type was not used

The probability of home deaths for older people who used any combination of LTC service type for home-dwelling recipients was higher compared with the 2.4% who did not use any service (Fig. 1). Those who used only in-home services had the highest probability (32.4%) of dying at home. Moreover, those who used day services or short-stay services with in-home services exhibited a higher probability of home death—21.4% with in-home services and day services, and 21.3% with in-home and short-stay services—than those who used only day services (15.3%) or short-stay services (3.0%).

A variation in IRR for each item was observed even within the same type of LTC service for home-dwelling recipients (Table 4). For example, within the type of in-home services, although the highest IRR was 3.80 (3.77–3.84) for renting welfare equipment, the last one was 0.85 (0.83–0.87) for rehabilitation. The results with covariates have been displayed in Additional Table 3.

**Table 4:** Association between home deaths and items in each LTC^a^ type for home-dwelling recipients^b^ (*n* = 2,035,657)

Service item in each type of LTC^c^	Incident rate ratios	Robust SE^d^	*p*-value	95% Confidential Interval	
No use	Reference				
In-home service for care	1.22	0.004	< 0.001	1.21	1.23
In-home service for nursing	1.29	0.005	< 0.001	1.28	1.30
In-home service for medical supports	1.77	0.007	< 0.001	1.76	1.78
In-home service for bath	1.75	0.007	< 0.001	1.74	1.77
In-home service for rehabilitation	0.85	0.009	< 0.001	0.83	0.87
In-home service for renting welfare equipment	3.80	0.020	< 0.001	3.77	3.84
Day service for care	1.19	0.005	< 0.001	1.18	1.20
Day service for rehabilitation	0.99	0.008	0.417	0.98	1.01
Short-stay service for care	0.91	0.005	< 0.001	0.90	0.92
Short-stay service for care with medical services	0.77	0.010	< 0.001	0.75	0.79

^a^ Long-term care

^b^ Poisson regression analysis, which was conducted to examine the association between the use of service item in each type of LTC for home-dwelling recipients and home death, adjusts for recipients’ age at time of death, gender, presence of spouse, underlying cause of death, year of death, degree of need for care, and secondary medical area dummy variables. Exponentiated coefficients were shown as incident rate ratios

^c^ Care recipients used service items in each type of LTC for home-dwelling recipients during/in the month of death

^d^ Standard errors

## Discussion

### Principal findings

This study assessed the association between the use of each type of LTC service for home-dwelling recipients, including their combined use, during/in the month of death and the recipients’ place of death. The use of every type of LTC service for home-dwelling recipients was positively associated with the recipients’ home death. These results suggest the important role of LTC service for home-dwelling recipients for supporting care recipients who wish to stay at home until their death as well as their informal caregivers. Moreover, those who received day services or short-stay services with in-home services shortly before their deaths were more likely to die at home as compared to those who used only day or short-stay services. Additionally, we discovered a heterogeneous association between the items within each type of LTC service for home-dwelling recipients and the probability of home death. Given the large gap between the ideal and reality as regards the place of death (i.e., at home versus at the hospital), our findings have many implications for how physicians, nurses, and care workers coordinate LTC services for home-dwelling recipients for older people who wish to stay at home until death.

### Possible mechanisms

There are some potential mechanisms through which the use of LTC services for home-dwelling recipients is associated with an increased probability of continuing care at home until death. First, the use of LTC services for home-dwelling recipients such as in-home services and day services may lead to a reduction in the psychological caregiving burden of recipients’ families and can help care recipients feel more secure and confident about their lives, by providing physical assistance with their ADL and IADL [35–39]. Second, as suggested in previous studies, LTC services for home-dwelling recipients such as day services and short-stay services—especially for patients with dementia and their caregivers—may sustain their cognitive function and improve their behavioral and psychological symptoms, which would enable them to live at home for a longer time [40–42]. Third, using formal LTC services for home-dwelling recipients may indicate that care recipients who wish to stay at home until death are taken care by informal caregivers because formal LTC services for home-dwelling recipients are often used to complement informal care, although the dummy variables of the presence of a spouse were adjusted. Conversely, if older persons are living alone, they will be less likely to use respite care services such as short-stay services. Living together with informal caregivers should be a significant driver of staying at home until death.

The association of short-stay services uses with home deaths might reflect complicated situations. On the one hand, short-stay services have been used as alternatives to hospitalization and residential services in Japan when hospital beds are unavailable and facility capacity is full [43–45]. In such cases, the recipients and their families wish to leave home. On the other hand, short-stay services, as a form of respite care, have reduced the care burden or psychological distress of caregivers [41, 46, 47]. A study in Australia reported that short-stay services improved the behavioral and psychological symptoms of dementia patients, [48] though the effectiveness of Japan’s short-stay services on recipients is yet unknown. Thus, our estimates for short-stay services might be contaminated by these mechanisms that have the opposite effects.

Furthermore, we found that people who received day or short-stay services with in-home services shortly before death were more likely to stay at home until death as compared to those who used only day or short-stay services. This might be because in-home services are essential for older adults who need support to live at home until death, even though day or short-stay services can give care recipients a chance of social activity and maintain their physical and psychological condition.

Moreover, we reported a heterogeneous association between the items of each type of LTC service for home-dwelling recipients with the probability of home death. The results suggest that the necessity of the items within each LTC service type for home-dwelling recipients is diverse for care recipients and their caregivers staying at home until death.

### Comparisons with previous studies

Several reviews have focused on the effects of the use of LTC services for home-dwelling recipients on care recipients’ ADL and caregiver burden [41, 49]. However, few studies on the association between the use of numerous LTC service types for home-dwelling recipients by care workers and place of death have been published despite the large gap between where dying people wish to stay until death and the place where they actually die. Most earlier studies have reported a positive association between in-home services provided by physicians and nurses with home deaths in Canada, Denmark, Japan, Taiwan, the United Kingdom, and the United States [10–13]; however, they did not examine the association between in-home services, day services, and short-stay services provided by non-medical professionals and the place of death regardless of the fact that medical care alone cannot reduce the family burden. Most recently, a research demonstrated a positive association between in-home service for care provided by care workers and home death, although it did not focus on LTC services for home-dwelling recipients other than in-home care services [14]. On the contrary, our study comprehensively examined the association of various LTC service types for home-dwelling recipients by non-medical care workers, including the combined use of such services, with the recipients’ home death. Moreover, we investigated the association of the items of each LTC service type for home-dwelling recipients and place of death for the first time, to our knowledge.

### Limitations

There were several limitations to the study. First, in the data management, the deterministic linkage with four identification variables was conducted to merge the Survey of Long-term Care Benefit Expenditures with the death records from Vital Statistics of Japan. Thus, there might be a possibility of false matching. In this study, as these administrative surveys included large population, a few missing variables, and a few misclassified identification variables, the deterministic linkage between them should have a high proportion of exact matches [50]. If the common variable at the individual level between two surveys is added in the future, we could conduct a more precise analysis.

Second, our estimates in the cross-sectional design at the time of death revealed an association between the use of each LTC service for home-dwelling recipients prior to the recipients’ death and the place of death. Thus, their effect on home deaths cannot be concluded. Furthermore, although many covariates were adjusted in this study, referring to the previous studies, unobserved confounders might have remained. In particular, the preferences of recipients and their informal caregivers regarding the place of death might confound between exposures and outcomes. We assumed that the variables of other LTC services’ use would work as a proxy for their preferences for the place of death, and that excluding the residents in LTC facilities and qualified nursing homes under LTCI from the study participants would ease this limitation. Since accurately measuring their preferences has been difficult, more studies using quasi-experimental and interrupted time series designs would be needed to deal with these unobserved confounders [51].

Third, in the study, home deaths might have included some unintentional and unattended deaths because such situations were not apparent in death records. However, we assumed that most recipients and their informal caregivers in this study were receiving appropriate medical and LTC services for home-dwelling recipients at the end of life because they had previously applied for LTCI, were using LTC services for home-dwelling recipients to some degree, and passed away with the exception of external cause of death.

## Conclusions

Our findings indicate that the use of LTC services for home-dwelling recipients by care workers at the end of life is associated with a higher probability of recipients’ dying at home. The probabilities of home deaths varied by the type and combination pattern of LTC services for home-dwelling recipients. Given the progressive aging around the world and the increasing need for people to die at the place where they wish to die, which is often at home, our findings can elucidate the importance of providing and combining LTC services for home-dwelling older people to support care recipients who wish to die at home and assist their informal caregivers.

## Supplementary information

**Additional file 1: Table 1.** Characteristics of long-term care beneficiaries aged 65 years and above who passed away in Japan between January 2008 and December 2013, by the use of each type of long-term care services for home-dwelling recipients.

**Additional file 2: Table 2.** Results for the association between the use of types of long-term care service for home-dwelling recipients and the recipients’ home death, using the Poisson regression analysis with covariates (*n* = 2,035,657).

**Additional file 3: Table 3.** Results for the association between the use of service items of each type of long-term care for home-dwelling recipients and the recipients’ home death, using the Poisson regression analysis with covariates (*n* = 2,035,657).

## Data Availability

The data that support the findings of this study are available from the Japanese Ministry of Health, Labour and Welfare, but restrictions apply to the availability of these data, which were used under license for the current study. Data are however available from the authors upon reasonable request and with permission of the Japanese Ministry of Health, Labour and Welfare.

## Abbreviations

LTC: Long-term care
LTCI: Long-term care insurance
IRR: Incident rate ratio
CI: Confidence interval
IADL: Instrumental activities of daily living
ADL: Activities of daily living
MHLW: Ministry of Health, Labour and Welfare
SE: Standard errors

## References

[1] Gomes B, Calanzani N, Gysels M, Hall S, Higginson IJ (2013). Heterogeneity and changes in preferences for dying at home: a systematic review. BMC Palliat Care.

[2] Higginson IJ, Sen-Gupta GJ (2000). Place of care in advanced cancer: a qualitative systematic literature review of patient preferences. J Palliat Med.

[3] Sadler E, Hales B, Henry B, Xiong W, Myers J, Wynnychuk L (2014). Factors affecting family satisfaction with inpatient end-of-life care. PLoS One.

[4] Cabinet Office Government of Japan (2012). The results of the survey on awareness of the elderly people about their health.

[5] Japanese Ministry of Health Labor and Welfare (2017). Trends in percent distribution of deaths by place of occurrence.

[6] Tamiya N, Noguchi H, Nishi A, Reich MR, Ikegami N, Hashimoto H (2011). Population ageing and wellbeing: lessons from Japan's long-term care insurance policy. Lancet..

[7] Japanese Ministry of Health Labour and Welfare (2017). The results of the survey on awareness of the elderly people about their health.

[8] World Health Organization (2017). The global strategy and action plan on ageing and health.

[9] World Health Organization (2015). World report on ageing and health 2015.

[10] Costa V, Earle CC, Esplen MJ, Fowler R, Goldman R, Grossman D (2016). The determinants of home and nursing home death: a systematic review and meta-analysis. BMC Palliat Care.

[11] Gomes B, Higginson IJ (2006). Factors influencing death at home in terminally ill patients with cancer: systematic review. BMJ..

[12] Takeu R (2008). A review of articles about factors association with death at home and death at hospital since 1990 in Japan. J Japan Acad Community Health Nurs.

[13] Gomes B, Calanzani N, Curiale V, McCrone P, Higginson IJ. Effectiveness and cost-effectiveness of home palliative care services for adults with advanced illness and their caregivers. Cochrane Database Syst Rev. 2013. 10.1002/14651858.CD007760.pub2. 10.1002/14651858.CD007760.pub2 PMC4473359 23744578

[14] Abe K, Miyawaki A, Kobayashi Y, Noguchi H, Takahashi H, Tamiya N (2019). Receiving the home care service offered by certified care workers prior to a patients’ death and the probability of a home death: observational research using an instrumental variable method from Japan. BMJ Open.

[15] Morioka N, Tomio J, Seto T, Yumoto Y, Ogata Y, Kobayashi Y (2018). Association between local-level resources for home care and home deaths: a nationwide spatial analysis in Japan. PLoS One.

[16] Izumida N. Relationship between improvement of in-home nursing care service and home death rate. IPSS Discussion Paper Series. 2012;2011(J05):1–30.

[17] Izumida N (2010). Analysis of the relationship between the difference in the place of death and the supply of medical and long-term care services. J Soc Secur Res.

[18] Japanese Ministry of Health Labour and Welfare (2017). Revision of the long-term care insurance act.

[19] Sakamoto H, Rahman M, Nomura S, Okamoto E, Koike S, Shibuya K, Gilmour S, Tatara K (2018). H Y. Japan health system review. Health systems in transition.

[20] Tsutsui T, Muramatsu N (2005). Care-needs certification in the long-term care insurance system of Japan. J Am Geriatr Soc.

[21] Japanese Ministry of Health Labour and Welfare (2017). In-home service for rehabilitation.

[22] Japanese Ministry of Health Labour and Welfare (2017). In-home service for medical supports.

[23] Japanese Ministry of Health Labour and Welfare (2017). In-home services for bath.

[24] Japanese Ministry of Health Labour and Welfare (2017). Day service for care.

[25] Japanese Ministry of Health Labour and Welfare (2017). Day service for rehabilitation.

[26] Japanese Ministry of Health Labour and Welfare (2017). In-home service for renting welfare equipment.

[27] Japanese Ministry of Health Labour and Welfare (2017). Short-stay services.

[28] Japanese Ministry of Health Labour and Welfare (2014). Residential services and in-home services.

[29] Japanese Ministry of Health Labour and Welfare (2013). Summary of results of the survey of medical institutions.

[30] Hospice Palliative Care Japan. Annual changes in the number of facilities and beds receiving notification of inpatient charges for palliative care wards. https://www.hpcj.org/what/pcu_sii.html. Accessed 23 Apr 2020.

[31] Japanese Ministry of Health Labour and Welfare (2016). List of statistical surveys conducted by Ministry of Health, Labour and Welfare.

[32] Japanese Ministry of Health Labour and Welfare. Table of ICD-10 (2003) codes on the cause of death. http://www.mhlw.go.jp/toukei/sippei/xls/shiin2003.xls. Accessed 9 Feb 2020.

[33] Barros AJD, Hirakata VN (2003). Alternatives for logistic regression in cross-sectional studies: an empirical comparison of models that directly estimate the prevalence ratio. BMC Med Res Methodol.

[34] Williams R (2012). Using the margins command to estimate and interpret adjusted predictions and marginal effects. Stata J.

[35] Tsutsui T (2010). Influence of the use of home services on family care burden of family caregiver. Home-Visit Nurs Nurs Care.

[36] Olaleye OA, Hamzat TK, Owolabi MO (2014). Stroke rehabilitation: should physiotherapy intervention be provided at a primary health care Centre or the patients' place of domicile?. Disabil Rehabil.

[37] Gjelsvik BE, Hofstad H, Smedal T, Eide GE, Naess H, Skouen JS (2014). Balance and walking after three different models of stroke rehabilitation: early supported discharge in a day unit or at home, and traditional treatment (control). BMJ Open.

[38] Roderick P, Low J, Day R, Peasgood T, Mullee MA, Turnbull JC (2001). Stroke rehabilitation after hospital discharge: a randomized trial comparing domiciliary and day-hospital care. Age Ageing.

[39] Hayasaka T, Mitadera Y (2003). Evaluation of home welfare services by the elderly person himself. J Health Welf Stat.

[40] Neville CC, Byrne GJ (2007). The impact of residential respite care on the behaviour of older people with dementia: literature review. Int J Older People Nursing.

[41] Vandepitte S, Van Den Noortgate N, Putman K, Verhaeghe S, Verdonck C, Annemans L (2016). Effectiveness of respite care in supporting informal caregivers of persons with dementia: a systematic review. Int J Geriatr Psychiatry.

[42] Zank S, Schacke C (2002). Evaluation of geriatric day care units: effects on patients and caregivers. J Gerontol B-Psychol.

[43] Japan Care Manager Association (2015). Report of the research on how to provide the respite care services that contribute to the continuation of living at home.

[44] Moriyama Y, Tamiya N, Kawamura A, Mayers TD, Noguchi H, Takahashi H (2018). Effect of short-stay service use on stay-at-home duration for elderly with certified care needs: analysis of long-term care insurance claims data in Japan. PLoS One.

[45] Kato G, Tamiya N, Kashiwagi M, Sato M, Takahashi H (2009). Relationship between home care service use and changes in the care needs level of Japanese elderly. BMC Geriatr.

[46] Lee D, Morgan K, Lindesay J (2007). Effect of institutional respite care on the sleep of people with dementia and their primary caregivers. J Am Geriatr Soc.

[47] Neville CC, Byrne GJ (2008). Effect of a residential respite admission for older people on regional Queensland family carers. Collegian..

[48] Neville CC, Byrne GJA (2005). The impact of residential respite care on the behavior of older people. Int Psychogeriatr.

[49] Maayan N, Soares-Weiser K, Lee H. Respite care for people with dementia and their carers. Cochrane Database Syst Rev. 2014. 10.1002/14651858.CD004396.pub2. 10.1002/14651858.CD004396.pub3 PMC11089513 24435941

[50] Zhu Y, Matsuyama Y, Ohashi Y, Setoguchi S (2015). When to conduct probabilistic linkage vs. deterministic linkage? A simulation study. J Biomed Inform.

[51] Zarit SH, Bangerter LR, Liu Y, Rovine MJ (2017). Exploring the benefits of respite services to family caregivers: methodological issues and current findings. Aging Ment Health.

