# Supplementary material for: Place of death associated with types of long-term care services near the end-of-life for home-dwelling older people in Japan: a pooled cross-sectional study
Source: BMC Palliat Care. 2020 Aug 9;19:121. doi: 10.1186/s12904-020-00622-0 (PMC7416406; doi:10.1186/s12904-020-00622-0)
Supplement: Supplementary file 1 — Additional file 1: Table 1. Characteristics of long-term care beneficiaries aged 65 years and above who passed away in Japan between January 2008 and December 2013, by the use of each type of long-term care services for home-dwelling recipients. [file 12904_2020_622_MOESM1_ESM.pdf]

Additional Table 1. Characteristics of long-term care beneficiaries aged 65 years and above who passed away in Japan between January 2008 and December 2013, by the use of each type of long-term care services provided for home-dwelling recipients

	Total (n=2035657)	No use (n=1098458)	Total of users* (n=937199)	In-home services (n=807785)	Day services (n=385573)	Short-stay services (n=148701)	In-home services and day services (n=280256)	In-home services and short-stay services (n=107377)	Day services and short-stay services (n=83338)	In-home, day service, and short-stay service (n=66111)
**Death by year (%)**										
**2008**	15.6	15.8	15.2	15.1	14.8	15.5	14.0	15.5	15.4	14.8
**2009**	15.6	15.8	15.3	15.2	15.1	15.7	14.7	15.7	15.8	15.4
**2010**	16.5	16.6	16.3	16.3	16.4	16.5	16.3	16.8	16.8	16.9
**2011**	17.2	17.2	17.3	17.3	17.6	17.1	17.7	17.2	17.4	17.5
**2012**	18.3	17.9	18.7	18.8	19.0	18.6	19.5	18.6	18.6	18.9
**2013**	16.9	16.7	17.1	17.3	17.2	16.5	17.8	16.2	16.1	16.5
**Place of death (%)**										
**Home**	13.9	2.3	27.5	29.8	21.1	20.4	23.1	25.2	23.0	25.2
**Other places†**	86.1	97.7	72.5	70.2	78.9	79.6	76.9	74.8	77.0	74.8
**Median (IQR‡) age, years**	85(79, 90)	84(79, 90)	85(79, 90)	85(79, 90)	86(81, 91)	88(79, 90)	86(81, 91)	88(83, 90)	88(83, 93)	88(83, 93)
**Gender (%)**										
**Male**	49.0	49.8	48.1	48.9	44.1	39.4	44.7	38.9	38.6	38.9
**Female**	51.0	50.2	51.9	51.1	55.9	60.6	55.3	61.1	61.4	61.1
**Degree of care need (%)**										
**Low**	8.6	9.0	8.2	6.7	9.6	1.4	5.1	0.7	1.4	0.6
**Moderate**	25.9	21.9	30.7	28.3	36.1	20.5	29.9	16.3	25.1	18.4
**High**	65.4	69.2	61.1	65.1	54.3	78.2	65.0	82.9	73.5	81.0
**Presence of spouse (%)**										
**Present**	44.6	44.4	45.0	46.3	38.2	32.9	38.8	33.6	31.9	33.1
**Unmarried**	3.2	3.6	2.7	2.8	2.3	2.0	2.5	1.7	1.6	1.5
**Bereavement**	48.1	47.6	48.7	47.1	56.1	62.7	55.2	62.4	64.4	63.3
**Divorce**	4.0	4.4	3.6	3.8	3.3	2.5	3.5	2.3	2.1	2.1
**Underlying cause of death (%)**										
**Cancer**	28.0	27.3	28.9	30.9	16.9	13.4	17.2	12.7	11.4	11.3
**Cardiovascular**	16.3	13.5	19.5	18.0	25.5	22.9	23.6	22.2	25.1	23.6
**Pneumonia**	13.0	15.1	10.5	10.2	12.4	15.2	12.8	15.1	14.7	14.9
**Cerebrovascular**	10.9	12.5	9.0	8.6	11.7	11.8	11.4	12.1	12.7	12.9
**Senility**	4.8	3.6	6.1	6.5	5.5	8.7	6.4	9.8	8.0	8.9
**Others**	27.1	27.9	26.1	25.9	28.0	28.0	28.6	28.2	28.1	28.4

* Long-term care (LTC) services were used by the recipients during/in the month of death. † Other places indicate hospitals, clinics with beds, LTC facilities, qualified nursing homes, and others such as day service facilities or outdoors ‡ Interquartile range.
