# Supplementary material for: Place of death associated with types of long-term care services near the end-of-life for home-dwelling older people in Japan: a pooled cross-sectional study
Source: BMC Palliat Care. 2020 Aug 9;19:121. doi: 10.1186/s12904-020-00622-0 (PMC7416406; doi:10.1186/s12904-020-00622-0)
Supplement: Supplementary file 2 — Additional file 2: Table 2. Results for the association between the use of types of long-term care service for home-dwelling recipients and the recipients’ home death, using the Poisson regression analysis with covariates (n = 2,035,657). [file 12904_2020_622_MOESM2_ESM.pdf]

Additional Table 2. Results for the association between the use of types of long-term care service for home-dwelling recipients and the recipients' home death, using the Poisson regression analysis with covariates[*] (n= 2035657)

	IRR[†]	Robust SE[‡]	p-value	95% Confidential Interval	
**Age**	1.012	<0.001	<0.001	1.011	1.012
**Gender**					
**Male**			Reference		
**Female**	1.034	0.004	<0.001	1.026	1.041
**Degree of care needed**					
**Low**	1.065	0.007	<0.001	1.051	1.078
**Moderate**	0.843	0.003	<0.001	0.837	0.850
**High**			Reference		
**Presence of a spouse**					
**Present**			Reference		
**Unmarried**	1.004	0.010	0.667	0.985	1.024
**Bereavement**	0.988	0.004	0.004	0.980	0.996
**Divorce**	0.999	0.009	0.894	0.981	1.017
**Underlying cause of death**					
**Cancer**	1.273	0.006	<0.001	1.261	1.285
**Cardiovascular**	1.853	0.009	<0.001	1.835	1.871
**Pneumonia**	0.512	0.005	<0.001	0.503	0.522
**Cerebrovascular**	1.428	0.009	<0.001	1.411	1.446
**Senility**	2.599	0.014	<0.001	2.572	2.627
**Others**			Reference		
**Year of death**					
**2008**			Reference		
**2009**	0.971	0.005	<0.001	0.961	0.982
**2010**	0.953	0.005	<0.001	0.943	0.963
**2011**	0.945	0.005	<0.001	0.935	0.955
**2012**	0.959	0.005	<0.001	0.949	0.969
**2013**	0.949	0.005	<0.001	0.939	0.959
**Use of types of long-term care services for home-dwelling recipients[§]**					

**No use**			Reference		
**In-home service for care**	13.398	0.089	<0.001	13.225	13.574
**Day service for care**	6.321	0.066	<0.001	6.193	6.452
**Short stay service for care**	1.249	0.045	<0.001	1.163	1.341
**Combination use of each type of long-term care services for home-dwelling recipients**[§]					
**In-home service and day service**[‖]	0.104	0.001	<0.001	0.102	0.106
**In-home service and short stay service**[‖]	0.526	0.020	<0.001	0.489	0.566
**Day service and short stay service**[‖]	0.721	0.030	<0.001	0.665	0.781
**In-home, day, and short-stay service**	2.136	0.091	<0.001	1.965	2.322

[*] Poisson regression analysis adjust for the dummy variables of secondary medical areas of care recipients' living municipality.

[†] Incident rate ratio.

[‡] Standard errors.

[§] The care recipients used long-term care services for home-dwelling recipients during/in the month of death. The other service type was not used.
