# Supplementary material for: Place of death associated with types of long-term care services near the end-of-life for home-dwelling older people in Japan: a pooled cross-sectional study
Source: BMC Palliat Care. 2020 Aug 9;19:121. doi: 10.1186/s12904-020-00622-0 (PMC7416406; doi:10.1186/s12904-020-00622-0)
Supplement: Supplementary file 3 — Additional file 3: Table 3. Results for the association between the use of service items of each type of long-term care for home-dwelling recipients and the recipients’ home death, using the Poisson regression analysis with covariates (n = 2,035,657). [file 12904_2020_622_MOESM3_ESM.pdf]

Additional Table 3. Results for the association between the use of service items of each type of long-term care for home-dwelling recipients and the recipients' home death, using the Poisson regression analysis with covariates* (n= 2035657)

	IRR†	Robust SE‡	p-value	95% Confidential Interval	
**Age**	1.008	<0.001	<0.001	1.007	1.008
**Gender**					
**Male**			Reference		
**Female**	1.005	0.004	0.190	0.998	1.013
**Degree of care need**					
**Low**	2.086	0.013	<0.001	2.060	2.113
**Moderate**	1.271	0.005	<0.001	1.261	1.281
**High**			Reference		
**Presence of spouse**					
**Present**			Reference		
**Unmarried**	1.078	0.011	<0.001	1.056	1.100
**Bereavement**	1.002	0.004	0.561	0.994	1.011
**Divorce**	1.050	0.010	<0.001	1.031	1.069
**Underlying cause of death**					
**Cancer**	1.328	0.006	<0.001	1.316	1.341
**Cardiovascular**	2.023	0.010	<0.001	2.003	2.043
**Pneumonia**	0.500	0.005	<0.001	0.490	0.509
**Cerebrovascular**	1.354	0.008	<0.001	1.337	1.370
**Senility**	2.450	0.014	<0.001	2.423	2.477
**Others**			Reference		
**Year of death**					
**2008**			Reference		
**2009**	0.949	0.005	<0.001	0.939	0.960
**2010**	0.920	0.005	<0.001	0.910	0.930

**2011**	0.899	0.005	<0.001	0.889	0.908
**2012**	0.898	0.005	<0.001	0.889	0.908
**2013**	0.872	0.005	<0.001	0.862	0.881
**Use of service item in each type of long-term care provided for home-dwelling recipients§**					
**No use**			Reference		
**In-home service for care**	1.218	0.004	<0.001	1.210	1.227
**In-home service for nursing**	1.292	0.005	<0.001	1.282	1.302
**In-home service for medical supports**	1.769	0.007	<0.001	1.755	1.783
**In-home service for bath**	1.754	0.007	<0.001	1.740	1.769
**In-home service for rehabilitation**	0.847	0.009	<0.001	0.829	0.865
**Renting welfare equipment**	3.803	0.020	<0.001	3.765	3.842
**Day service for care**	1.188	0.005	<0.001	1.178	1.198
**Day service for rehabilitation**	0.994	0.008	0.417	0.979	1.009
**Short-stay service for care**	0.913	0.005	<0.001	0.903	0.923
**Short-stay service for care with medical services**	0.769	0.010	<0.001	0.750	0.789

* Poisson regression analysis adjust for the dummy variables of secondary medical areas of care recipients' living municipality.
† Incident rate ratio.
‡ Standard errors.
§ The care recipients used service items in each type of long-term care service for home-dwelling recipients during/in the month of death.
